# Activated Olive Stones as a Low-Cost and Environmentally Friendly Adsorbent for Removing Cephalosporin C from Aqueous Solutions

**DOI:** 10.3390/ijerph18094489

**Published:** 2021-04-23

**Authors:** Gerardo León, Francisco Saura, Asunción María Hidalgo, Beatriz Miguel

**Affiliations:** 1 Department of Chemical and Environmental Engineering, Technical University of Cartagena, Paseo Alfonso XIII, 30203 Cartagena, Spain; franciscosauramoreno@hotmail.com (F.S.); beatriz.miguel@upct.es (B.M.); 2 Department Chemical Engineering, Campus of Espinardo, University of Murcia, 30100 Murcia, Spain; ahidalgo@um.es

**Keywords:** biosorption, agricultural wastes, emerging pollutants, equilibrium, kinetics, thermodynamics

## Abstract

In this paper, we describe the removal of cephalosporin C (CPC) from aqueous solutions by adsorption onto activated olive stones (AOS) in a stirred tank. For comparative purposes, several experiments of adsorption onto commercial granular activated carbon were carried out. A quantum study of the different species of cephalosporin C that, depending on the pH, exist in aqueous solution pointed to a favorable mass transfer process during adsorption. Activated olive stones were characterized by SEM, EDX and IR techniques and their pH_zc_ was determined. A 10^−3^ M HCl cephalosporin C solution has been selected for the adsorption experiments because at the pH of that solution both electrostatic and hydrogen bond interactions are expected to be established between the adsorbate and the adsorbent. The adsorption process is best described by the Freundlich isotherm model and the pseudo-second-order kinetic model, while the adsorption mechanism is mainly controlled by film diffusion. Under the conditions studied, the adsorption process is of a physical nature, endothermic and spontaneous. Comparison of the adsorption results obtained in this paper with those of other authors shows that the efficiency of AOS is 20% of that of activated carbon but 65% higher than that of the XAD-2 adsorbent. Considering its low price, abundance, easy accessibility and eco-compatibility, the use of activated olive stones as adsorbents for the removal of emerging pollutants from aqueous solutions represents an interesting possibility from both the economic and the environmental points of view.

## 1. Introduction

Pollution, one of the most important environmental problems that affect our world, is the result of the introduction of any substance into the environment in such a quantity that it causes adverse effects on living beings, subjecting them to doses that exceed acceptable level [1].

Pollution can come from certain manifestations of nature (natural sources) or from the myriad of production processes generated by man (anthropogenic sources). The sources that generate pollution of anthropogenic origin are, fundamentally, of an industrial, commercial, agricultural or domestic origin.

Evaluation of the impact of water pollution by chemical products has traditionally focused almost exclusively on the so-called priority pollutants, substances that present a significant risk to the aquatic environment or human health, as they are toxic, persistent or bioaccumulative. However, in recent decades, various chemical and biological agents have been increasingly detected in ecosystems and whose potential toxicity for health and the environment is of growing concern—theso called emerging pollutants. The term emerging pollutants refers to new contaminants identified in aquatic environments and organisms or to new characteristics and impacts of compounds that are already present in the environment. Norman Network (2016) has defined emerging pollutants as substances detected in the environment but which are not currently included in routine environmental monitoring programs and which may be the subject of future legislation due to their adverse effects [2]. They include a wide variety of compounds, including drugs, personal care products, illicit drugs, sweeteners, industrial additives and agents, flame retardants, surfactants, etc.

Drugs are probably the most studied emerging pollutants, both for their environmental relevance and for their effects. They include a diverse group of chemical products along with their metabolites and transformation products, which are incorporated into the environment during their industrial manufacturing processes and through their clinical and domestic applications [3,4].

Antibiotics (antimicrobials or antibacterial agents) are one of the most widely used categories of pharmaceutical products, with human, veterinary and agricultural applications [5]. They may be natural, synthetic or semisynthetic compounds, which can kill or inhibit the growth or metabolic activity of microorganisms. Different types of antibioticsare classified according to their action mechanism, mode of administration, source, spectrum of action, and chemical structure [6].

Due to the difficulty of removing these compounds in traditional wastewater treatment plants, large amounts of antibiotics have been transferred from industrial and domestic effluents into surface water, groundwater and even drinking water [7]. The presence of residual antibiotics in water and soil ecosystems is the main concern because these pollutants induce multi-resistance in bacteria that have dangerous health effects in aquaculture, humans, agriculture and livestock [8].

**Figure 1:**
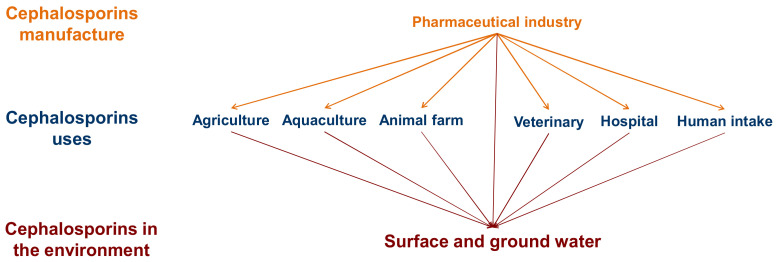
Sources of the presence of cephalosporins in the environment.

Cephalosporins are a group of broad-spectrum antibiotics that act by inhibiting the biosynthesis of the cell wall (peptidoglycan layer) of bacterial organisms [9]. The global market for cephalosporins was valued at USD 13.69 billion in 2019 and is estimated to reach USD 16.87 billion by 2027 [10]. They are commonly applied in humans, veterinary medicine, and aquaculture, making their clinical production and consumption a huge industry. As a consequence, there has been a significant increase in the presence of these substances in the environment, as many studies have shown [11,12] (Figure 1).

**Figure 2:**
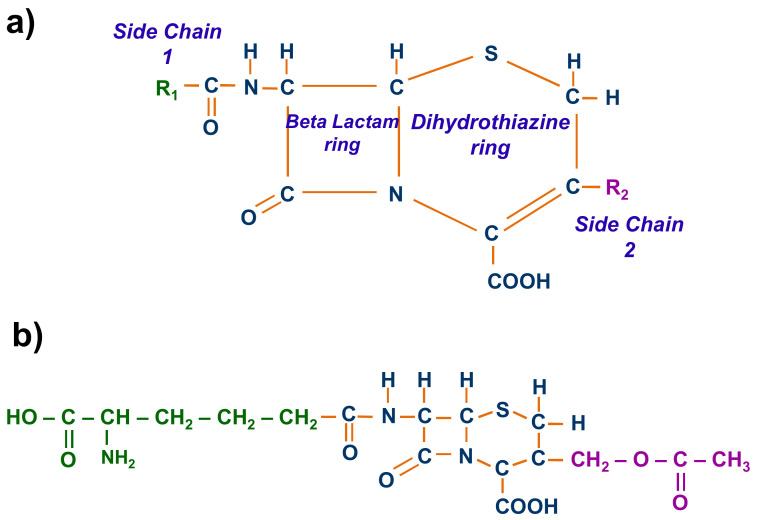
(**a**) General chemical structure of cephalosporins; (**b**) chemical structure of cephalosporin C.

Cephalosporin molecules are composed of a central structure, a beta-lactam ring attached to a dihydrothiazine ring and two main substituents (Figure 2a) [13]. The beta-lactam ring and the acylamido side chain 1 are responsible for the antibacterial activity, while side chain 2 primarily governs the pharmacokinetics [14].

Many studies have been published that describe different methods for removing cephalospirins from aqueous media, most of them related with technologies such as biological [15,16], adsorption [17,18] and oxidation process [19,20].

Adsorption is one of the most effective, economic and efficient methods for removing pollutants. Its many advantages include cost-effectiveness, ease of operation and applicability in continuous and discontinuous processes [21,22]. Activated carbon is the most frequently used adsorbent because of its high porosity, high specific surface area, and presence of a variety of surface groups [23], but interest in the use of adsorbents derived from agricultural residues has notably increased in recent years [24,25].

This paper studies the application of such agricultural residues as an adsorbent for the elimination of cephalosporins. Cephalosporin C, an intermediate in the manufacture of semisynthetic cephalosporin antibiotics, was selected as the target contaminant (Figure 2b). Activated olive stones, a by-product of the production of olive oil and table olives, were used as adsorbent, due to their significant adsorption properties, low cost (if any), abundance, ready availability (as a result of their production at the local level), good mechanical and chemical resistance andeco-compatibility at large [26]. Although olive stones, both raw and activated, have been used as an adsorbent to remove different metals, there are no studies of their application to remove emerging contaminants.

## 2. Materials and Methods

### 2.1. Materials

Cephalosporin C and activated carbon Darco AC 4–12 were obtained from Sigma-Aldrich. NaOH and HCl 36% were purchased from Panreac. Crushed raw olive stones (size ranging from 1 to 4 mm) were supplied by the olive-oil extraction plant Almazara Valle de Ricote, located in Archena, Murcia (Spain).

### 2.2. Methods

#### 2.2.1. Cephalosporin C Quantum Calculations

Theoretical calculations were carried out to investigate the molecular properties of the theoretical uncharged cephalosporin molecule (Figure 1) and compare the same with the properties of its five ionic forms in aqueous solution. The molecular structures were optimized at Hartree-Fock DFT level (HF-DFT) using the hybrid B3LYP functional [27,28] with the 6–31G* basis set, using the Gaussian 03 program [29]. Moreover, the dielectric polarizable continuum model (PCM) [30] was used to model the solvation effects of the water solvent, i.e., modeling the solvent as a polarizable continuum. The singlet and triplet states of all the ions were computed in order to discern the more stable state. Previous studies of beta-lactam derivatives [31] have been proved to be very useful in the study of reactivity and molecular properties of these compounds.

#### 2.2.2. Adsorbent Preparation

Crushed olives stones were washed with water and dried a at 60 °C in an oven before they were subjected to the activated process. The thermal treatment was performed by heating the samples at 300 °C in a closed muffle furnace to increase the surface area [32]. The sorbent material was then treated with 1 M HCl at room temperature (25 ± 2 °C) for 8 h to eliminate soluble components of olive stones (tannins, resins, reducing sugars and coloring agents), increment their active surface and generate oxygen functional groups such aslactones [33,34]. Finally, the thermal-acid activated olive stones (AOS) were filtered, washed abundantly with ultrapure water, dried at 100 °C for 24 h and stored in a desiccators.

Commercial activated carbon (AC), used as reference adsorbent, was washed several times with deionized water to eliminate powder carbon, dried in an oven at 105 °C for 24 h and stored in a desiccators.

#### 2.2.3. Adsorbent Characterization

The outer surface and the elemental composition of AOS before and after adsorption of CPC were analyzed by scanning electron microscopy (SEM) and energy-dispersive X-ray spectroscopy (EDX) [35], using a SEM HITACHI S-3500N apparatus, containing secondary and backscattered electron detectors (Hitachi High-Technologies Corporation, Tokyo, Japan), equipped with an EDX XFlash 5010 analysis system (Brukers AXS, Karlsruhe, Germany). Using 15 kV and a 10 mm work distance, samples were sputtered with a thin layer of platinum during 90 s by a sputter coater Polaron SC 7640 (Quorum Technologies, Newhaven, UK) and 5000× magnification wasused in the SEM study, while 15 kV and a 15 mm work distance were used in EDX analysis.

The point of zero charge (pH_pzC_), the pH at which the adsorbent is neutral in aqueous suspension, was determined, essentiallyaccording to the following procedure [36]. Aliquots of 50 mL NaCl (0.1 M) solutions were poured into conical flasks. The pH of each flask was adjusted from 2 to 10 by adding either HCl (0.1 M) or NaOH (0.1 M) solutions (pH_i_), using a Crison Basic 20 pH-meter. Then, 0.1 g of activated olive stones was added to each flask, which were shaken for 24 h before measuring the final pH (pH_f_). The difference between pH_i_ and pH_f_ (ΔpH) was plotted against pH_i_, the point of zero charge being the initial pH value at which ΔpH = 0.

Infrared spectrometry (IR) was used to identify changes in the chemical functional groups of the outer surface of both raw and activated olive stones [37]. A NICOLET 5700 FTIR apparatus (ThermoFischer Scientific, Waltham, MA, USA) was used in transmittance mode from 400 to 4000 cm^−1^.

#### 2.2.4. Adsorption Experiments

Batch adsorption experiments were carried out in a thermostated rotary shaker using a set of 50 mL Erlenmeyer flasks containing 40 mL of CPC solution of different concentrations (100–400 mg/L) in HCl 10^−3^ M and 0.100 g of AOS at 200 rpm, for 24 h at temperatures ranging from 10 to 40 °C. Samples were taken at different predetermined times and, after the addition of 1 M HCl, the CPC concentration in the solution was measured by UV–V spectrophotometry, at 508 nm, using an Agilent 8453 spectrophotometer. The CPC concentration in an unknown sample was determined from the calibration curve (concentration range: 100–500 mg/L; R^2^ = 0.9989). The results obtained showed a maximum deviation of 4%.

The amount of CPC loaded onto the AOS at any time t, q_t_ (mg/g), and at equilibrium, q_e_ (mg/g), was estimated from the following relationships [38]:

(1) $$q_{t}=\left(C_{0}-C_{t}\right)\cdot \frac{V}{m}$$

(2) $$q_{e}=\left(C_{0}-C_{e}\right)\cdot \frac{V}{m}$$

where C_0_, C_e_ and C_t_ are the initial, equilibrium and time t concentrations of CPC in the solution (mg/L), V is the volume of CPC solution (L), and m is the mass of adsorbent (g). Data obtained from these experiments were used to test the different equilibrium, kinetic, and adsorption mechanism models and to obtain the thermodynamic parameters.

## 3. Results and Discussion

### 3.1. Cephalosporin C Quantum Calculations

**Figure 3:**
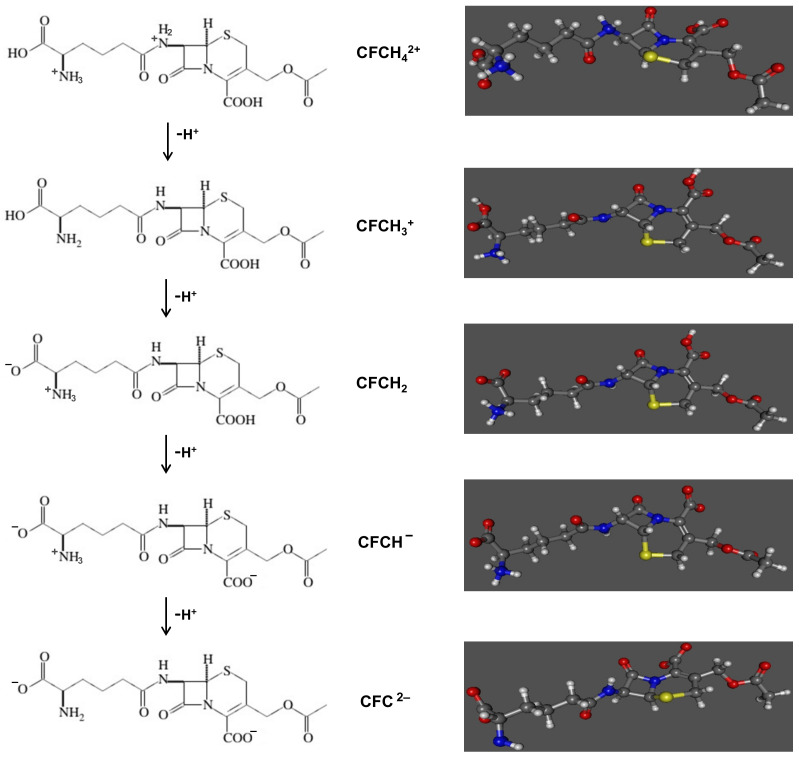
Representation of the different chemical structures of cephalosporin C in aqueous solution.

In aqueous solution, cephalosporin C can be present in different chemical forms, according to the pK_a_ values of its protonated functional groups [39,40,41,42] and depending on the pH of the solution (Figure 3).

From the optimized geometries of the molecular species, it can be seen that the atomic distances are quite similar, the highest differences being 0.05 Angstrom. However, there were several differences in the geometrical distribution of the beta lactam ring with respect to the amino group. These differences would be due to the protonation of the nitrogen, which forces the angle between the nitrogen and the two adjacent carbons (referred to in Table 1 as A(11,14,13) and A(11,14,15)) leading to a variation that ranges between 2 and 4 degrees, respectively. Moreover, the dihedral angles (referred to in Table 1 as D(11,14,13,2), D(11,14,13,10) and D(13,14,15,2)) between atoms in the amino group and the ring varied by between 5 and 3 degrees. These variations are due to the N-H bond as a result of changing hybridization on N from sp^2^ to sp^3^ [43].

The mass transfer that takes place during the adsorption process depends on the diffusion of the solute from the aqueous phase to the surface of the adsorbent, the diffusion of the adsorbate molecules into the pores of the adsorbent, and the adsorption of the molecules of solute on the surface. The first factor depends on the concentration gradient, and the other two depend on the size of the solute molecule and adsorbate–adsorbent molecular interactions. The molecular interactions will depend on the distribution of the molecular charge density and therefore the molecular dipole.

The Mulliken charge analysis shows the charge differences expected as a function of the molecular charge, in the oxygen atoms belonging to the -COO^-^ groups, and in nitrogen in the amine and amide groups, as shown in Table 2. Moreover, the molecular dipole, molar volume and total non-electrostatic interaction are shown in the Table 2.

The dipole moment of ions is higher than that of the cephalosporin theoretical molecule due to the electrical charges of the molecule. Therefore, the interactions due to the forces of the Waals path will favor the adsorption of the ions on the surface of the activated olive stones.

The chemical reactivity of the molecules is related to the difference in energy between the higher-energy occupied molecular orbital (HOMO) and the lower-energy unoccupied molecular orbital (LUMO). The electron density of HOMO is related to the electron donating capacity of the molecule and the electron accepting capacity of the molecule is associated with the availability of the electrons to access the LUMO, that is, the tendency of the molecule to accept electrons. The values obtained for these energy gaps were similar for the molecular species studied, so the availability to participate in electron transfer processes was similar for all of them, and in all cases the effect was favorable.

The size and molecular geometry are other factors that contribute to the adsorption of molecules on the porous surface of activated olive stones. As we have seen above, the difference between the geometry of cephalosporin and its ions would not justify great differences in the adsorption process.

The shorter-range non-electrostatic effects such as cavitation, dispersion and solvent structural effects, included in the total non-electrostatic energy, reflect both the hydrogen bonding and exchange repulsion effects between the molecule and the solvent.This non-electrostatic energy varied between 0.80 and 0.86 eV, confirming the stabilization of the ions in solution.

In summary, from the quantum study of the different species of cephalosporin C existing in aqueous solutions depending on the pH, it can be concluded that molecular size, adsorbate–adsorbent molecular interactions, dipole moment and chemical reactivity predicted by the molecular orbitals show a favorable mass transfer process during adsorption.

### 3.2. Adsorbent Characterization

**Figure 4:**
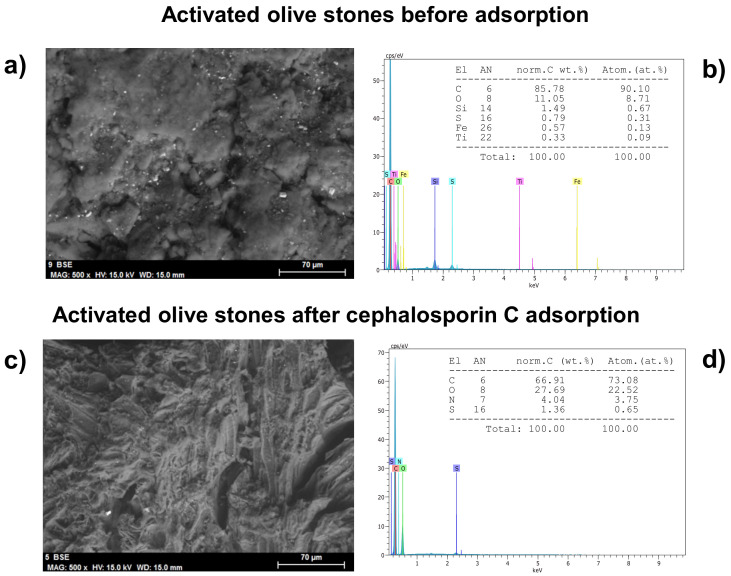
SEM (**a**,**b**) and EDX (**c**,**d**) of AOS before and after the adsorption of CPC.

SEM showed the surface microstructure of AOS before and after the adsorption of cephalosporin C in terms of the surface morphology and porous structure (Figure 4a,c). The surface of activated olive stones changed significantly after Cephalosporin C adsorption to be uneven, rough, undulating and with no perceptible pores.

EDX characterization pointed to the absence of nitrogen and sulfur in AOS (Figure 4b), but their presence in this adsorbent after cephalosporin C adsorption (Figure 4d).

Both facts confirm the adsorption of cephalosporin C onto the activated olive stones.

**Figure 5:**
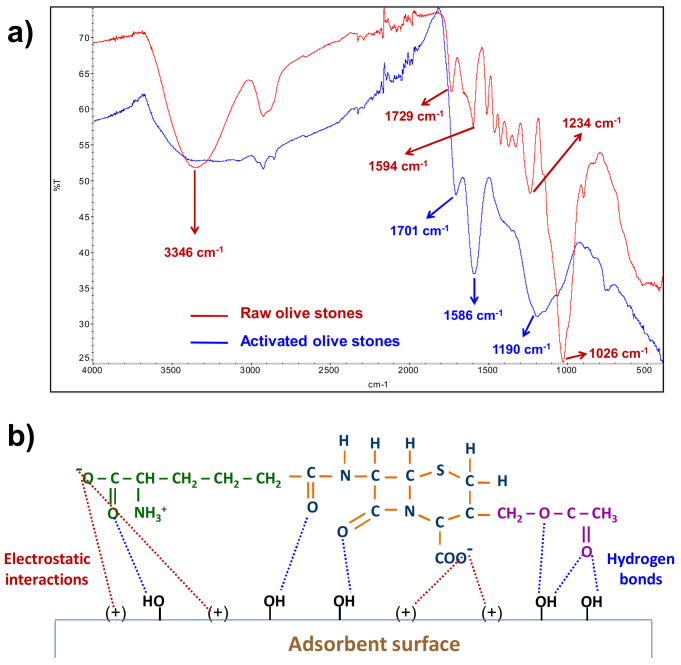
(**a**) FTIR spectra of raw and activated olive stones; (**b**) interactions between AOS and CPC.

Analysis of the FTIR of both raw and activated olive stones (Figure 5a) showed that the activation process led to a significant modification of the functional groups on the surface of the adsorbent. The intense –OH st band at 3346 nm, corresponding to the hydroxyl functional group of the main components of raw olive stones (cellulose, hemicellulose and lignin), was turned into a very broad absorption band between 3000 and 3600 nm corresponding to the –OH st band of the carboxylic, phenolic and hydroxyl functional groups, the width of this band indicating the presence of strong hydrogen bonds [37,44]. In addition, the very intense C–O st absorption band at 1026 of alcoholic hydroxyl [44,45] had decreased and the three intense bands (1701, 1586 and 11,904 cm^−1^) corresponding to carbonyl groups of different functional groups (ketone, lactone and carboxylic acid [44,45,46] had appeared. This agrees with the described presence of carboxylic, phenolic, lactone and carbonyl groups in H_3_PO_4_/thermal activated olive stones [47,48].

The point of zero charge determines the surface charge of the adsorbent at a given pH and supplies information about the possible electrostatic interactions with the adsorbate [49]. The point of zero charge of the AOS was found to be 3.5, which means that it is an acidic adsorbent and that the thermal/acid activation process provides some acidic groups to the AOS surface. At pH values lower than 3.5, the AOS surface will have a net positive charge, at pH values higher than 3.5, the surface will have a net negative charge and at pH = 3.5, it will have a net zero charge as a consequence of the presence of an equal number of both positive and negative charges.

The ionization of the protonated amide group and the two carboxyl groups of cephalosporin C is completed at a pH slightly lower than 3 and the deprotonation of the positive amine group takes place at basic pH [39,40,41,42], which means that at pH = 3, cephalosporin C has a negative net charge.

Since at pH 3 activated olive stones have a positive net charge and cephalosporin C has a negative net charge, HCl 10^−3^ M was the experimental medium selected in the adsorption tests, since at the pH of that medium both electrostatic interactions between the oppositely charged groups of adsorbent and adsorbate, and hydrogen bond interactions between the hydroxyl groups on the adsorbent surface and the carbonyl groups of the cephalosporin C molecule can be established (Figure 5b).

### 3.3. Equilibrium Studies

An adsorption isotherm represents an equilibrium relationship between the amount of adsorbate in the liquid phase and that on the adsorbent surface, at a given temperature. In this study, the experimental results of equilibrium were adjusted to six adsorption isotherms models (Langmuir, Freundlich, Elovich, Temkin, Javanovic and Dubinin–Radushkevich) in order to determine which model best describes the adsorption of CPC on AOS.

The Langmuir isotherm model assumes a monolayer adsorption, homogeneous distribution of the adsorption sites, constant adsorption energy and negligible interaction between adsorbate molecules [50,51]. The Hanes–Woolf linearization of the Langmuir isotherm can be written as [52]:

(3) $$\frac{C_{e}}{q_{e}}=\frac{C_{e}}{q_{m}}+\frac{1}{q_{m}\cdot K_{L}}$$

where q_m_ is the maximum monolayer adsorption capacity of the adsorbent (mg/g) and K_L_ is the Langmuir adsorption constant (L/mg). The values of q_m_ and K_L_ were calculated from the slope and the intercept of the plot of C_e_/q_e_ versus C_e_.

To confirm the favorability of the process, the dimensionless equilibrium parameter R_L_ is used, which is defined as:

(4) $$R_{L}=\frac{1}{1+K_{L}\cdot C_{0}}$$

where C_0_ refers to the highest initial cephalosporin C concentration in solution. The adsorption nature is indicated by R_L_ value either to be irreversible (R_L_ = 0), favorable (0 < R_L_ < 1), linear (R_L_ = 1) or unfavorable (R_L_ > 1) [53].

The Freundlich model is employed to describe a multilayer adsorption process on a heterogeneous surface with a non-uniform distribution of adsorption heat and affinities [54,55]. Its linear form is expressed as [55]:

(5) $${\mathrm{lnq}}_{e}=\frac{1}{n}\cdot {\mathrm{lnC}}_{e}+{\mathrm{lnK}}_{F}$$

where K_F_ and n are constants integrating all factors affecting the adsorption capability and adsorption intensity, respectively. The plot of lnq_e_ versus lnC_e_ allows the values of n and K_F_ to be determined from the slope and the intercept, respectively.

The parameter 1/n confirms the favorability of the process: irreversible (1/n = 0), favorable (0 < 1/n < 1), unfavorable (1/n > 1) [56].

The Elovich isotherm model suggests a multilayer adsorption in which the number of adsorption sites increases exponentially with adsorption. The linear form of the Elovich isotherm can be written as [57]:

(6) $$\ln\frac{q_{e}}{C_{e}}={\ln(K}_{E}\cdot q_{\mathrm{mE}})-\frac{q_{e}}{q_{\mathrm{mE}}}$$

where q_mE_, maximum Elovich adsorption capacity (mg/g), and K_E_, Elovich equilibrium constant (L/mg), can be calculated from the slope and intercept of the plot of ln(q_e_/C_e_) versus q_e_.

The Temkin isotherm model considers that the adsorption energy (ΔH) decreases linearly with coverage due to adsorbate–adsorbent interactions [58,59], and it can be represented in its linearized form by [57]:

(7) $$q_{e}=B\cdot \mathrm{lnA}+B\cdot {\mathrm{lnC}}_{e}$$

where A (L/mg) is the adsorption equilibrium constant, B is the Temkin constant, related to the heat of adsorption (B = q_m_·R·T/ΔH, mg/g). The values of B and A can be calculated from the slope and the intercept of the plot of q_e_ versus lnC_e_.

The Javanovic model suggests multilayer adsorption on a heterogeneous surface but considering the mechanical contacts between the adsorbed and solution phases and in its linear form can be expressed as [60,61]:

(8) $${\mathrm{lnq}}_{e}={\mathrm{lnq}}_{m}-K_{J}{\cdot C}_{e}$$

where q_m_ (mg/g) is the maximum adsorption capacity and K_J_ is the Javanovic constant (L/mg). From the plot of lnq_e_ versus C_e_, the values of the parameters of the model can be obtained.

The Dubinin–Radushkevich model assumes that the distribution of pores in a heterogeneous surface of the adsorbent follows a Gaussian energy distribution and it is usually applied to distinguish between physical and chemical adsorption processes [62,63].

The linearized form of the Dubinin–Radushkevich model is represented by:

(9) $${\mathrm{lnq}}_{e}={\mathrm{lnq}}_{m}-K\cdot \varepsilon^{2}$$

where q_m_ (mg/g) is the maximum adsorption capacity, K (mol^2^/J^2^) is a constant related to the sorption energy and ε (J/mol) is the adsorption potential, which can be calculated as follows:

(10) $$\varepsilon =\mathrm{RTln}\left(1+\frac{1}{C_{e}}\right)$$

Values of K and q_m_ can be obtained from the slope and the intercept of the plot of lnq_e_ versus ε^2^. The mean free energy of adsorption, E (J/mol), can be calculated from the constant K by the equation E = (2·K)^−1/2^, and it is frequently applied to determine whether the adsorption mechanism is dominated by physical interactions (E < 8 KJ/mol), ion exchange (8 KJ/mol < E < 16 KJ/mol) or chemical bonds (E > 20 KJ/mol) [63].

**Figure 6:**
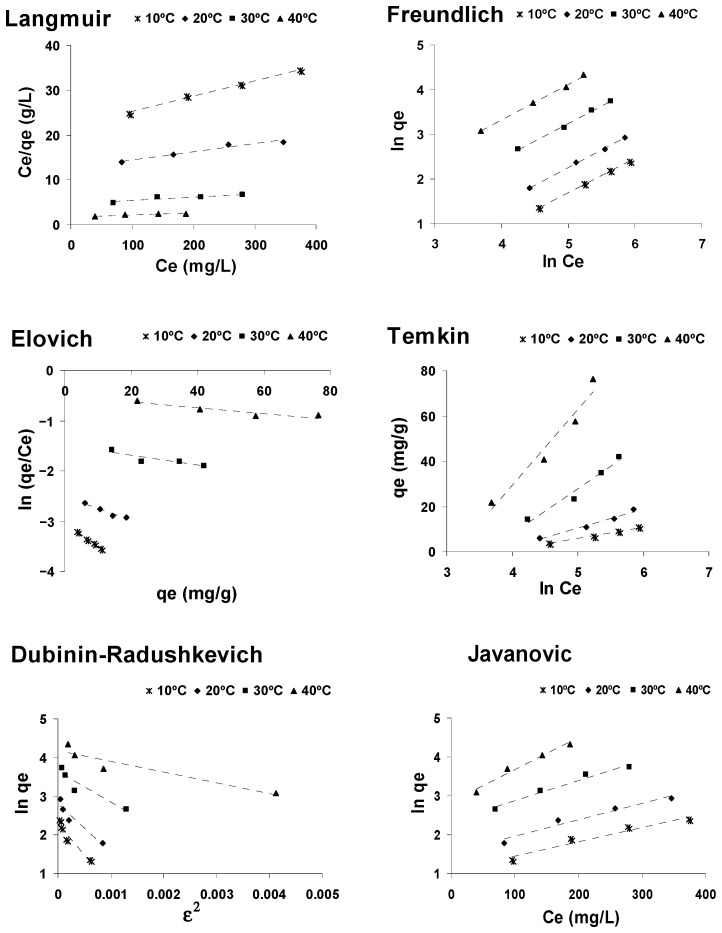
Isotherm plots for the adsorption of CPC onto AOS.

Linear regression was used to determine the isotherm that best defines the adsorption of CPC on activated olive stones. The corresponding linear regressions were carried out by comparing the correlation coefficients obtained. Figure 6 shows the linear representation of the six models described above, while the values of the characteristic constants of these models and of the R^2^ values obtained for each of them are included in Table 3.

The Freundlich isotherms are linear over the whole temperature range studied and the correlation coefficients (R^2^) were higher than those of the Langmuir, Elovich, Temkin, Javanovic and Dubinin–Radushkevich isotherms, indicating that the Freundlich isotherm best represented the experimental adsorption data of cephalosporin C onto activated olive stones, at all the studied temperatures. The fit of the experimental data to the Freundlich isotherm model suggests that the surface of activated olive stones is made up of homogeneous activated patches and the calculated values of 1/n confirm the favorable character of the adsorption process.

Although the Dubinin–Radushkevich model does not fit well with the experimental data, the adsorption mean free energy parameter of this model has been calculated, obtaining a value lower than 8, which suggests the physical character of the adsorption process.

### 3.4. Kinetic Studies

#### 3.4.1. Adsorption Kinetics

The adsorption kinetics describe the rate of retention of an adsorbate from a fluid environment onto an adsorbent, determining the time required to reach equilibrium.

The kinetics of CPC adsorption onto AOS were studied by means of four kinetic models (Lagergren pseudo-first order, Ho pseudo-second order, Elovich and Avrami) in order to determine the rate constant of the one that best describes the adsorption process of cephalosporin C onto activated olive stones.

The Lagergren pseudo-first-order model [64,65] is based on the assumption that the rate of occupation of sorption sites is proportional to the number of unoccupied sites and is expressed linearly by the following equation:

(11) $$\ln\left(q_{e}-q_{t}\right)={\mathrm{lnq}}_{e}-k_{\mathrm{ps}1}\cdot t$$

where k_sp1_ (1/min) is the pseudo-first-order adsorption rate constant. The representation of ln (q_e_ − q_t_) versus time allows the values of the model constants, k_sp1_ and theoretical q_e_, to be determined from the intersection and the slope, respectively.

The Ho pseudo-second-order kinetic model [66] assumes that adsorption implies the interaction between the adsorbate and two independent unoccupied sites on the adsorbent material [67]. In its linear form, it can be represented by the following equation:

(12) $$\frac{t}{q_{t}}=\frac{1}{k_{\mathrm{ps}2}\cdot q_{e}^{2}}+\frac{t}{q_{e}}$$

where k_ps2_ (L/mol·min) is the pseudo-second-order adsorption rate constant and the product k_ps2_·q_e_^2^ is the initial adsorption rate. From the representation of t/q_t_ versus time, the model constants, k_ps2_ and theoretical q_e_, can be obtained from the intercept and the slope, respectively.

The Elovich model [68] considers that the rate of adsorption of the solute decreases exponentially as the amount of adsorbed solute increase. Assuming that α·β·t >> 1 [69], this model can be expressed in a linear way using the following equation:

(13) $$q_{t}=\frac{\ln\left(\alpha \cdot \beta \right)}{\beta }+\frac{\mathrm{lnt}}{\beta }$$

where α (mg/g·min) is the initial adsorption rate and β (g/mg) is a constant related to the number of sites available for adsorption. By plotting q_t_ versus lnt, the model constants (β and α) can be obtained from the slope and the intercept, respectively.

The Avramy kinetic model [70] assumes that the solute–solvent interaction is located at the active sites on the surface of the solid support. In its linear form, it is described by the following double logarithmic expression [71]:

(14) $$\ln\left[-\ln\left({1-q}_{t}\right)\right]={\mathrm{lnK}}_{\mathrm{av}}+n_{\mathrm{av}}\cdot \mathrm{lnt}$$

where K_av_ is Avrami’s constant rate (min^−1^) and n_av_ is Avrami’s order model.

The kinetics of cephalosporin C adsorption on activated olive stones at 20 °C were analyzed using the above-mentioned kinetic models. The reliability of the fit of the different models to the experimental data was determined by comparing the values of the correlation coefficient (R^2^) and the degree of coincidence of the experimental values of q_e_ with the theoretical values calculated from the model.

**Figure 7:**
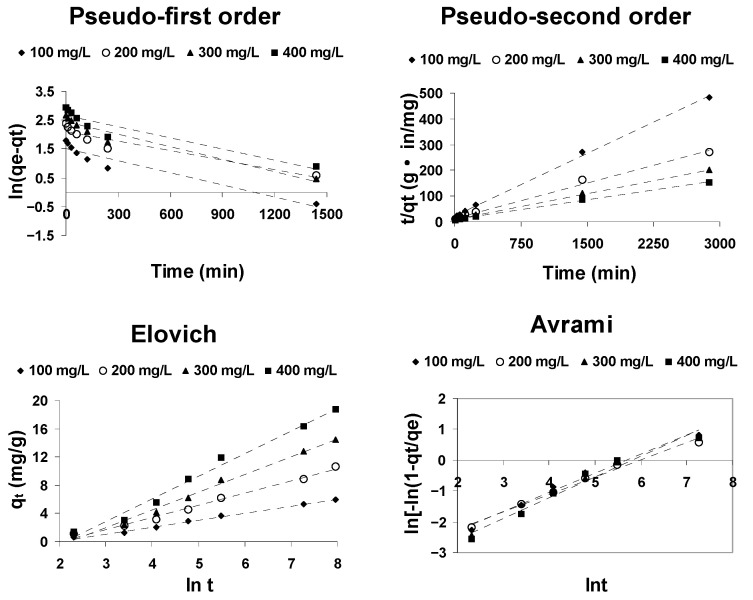
Kinetic plots for the adsorption of CPC onto AOS at 20 °C.

As it is evident from the results shown in Figure 7, the adsorption of cephalosporin C onto activated olive stones is best fitted by the pseudo-second-order kinetic model. Table 4 shows that the correlation coefficient R^2^ for the pseudo-second-order kinetic equation is greater than 0.99 and that the calculated q_e_ values are acceptably close to the experimental values, which confirms that the pseudo-second-order kinetic model adequately describes the adsorption of cephalosporin C onto activated olive stones. By contrast, the correlation coefficients corresponding to the pseudo-first-order, Elovich and Avrami kinetic models are significantly lower than 0.99, which means that none of these models is suitable to describe the adsorption of cephalosporin C in activated olive stones.

#### 3.4.2. Adsorption Mechanism

The kinetic models described above are not capable of identifying the rate-controlling step of the adsorption process. Any solid–liquid adsorption process is generally characterized by extraparticle diffusion (boundary layer diffusion), by intraparticle diffusion, or by both [72]. To identify the rate-controlling step of the adsorption process, the Weber and Morris intraparticle diffusion model and the Boyd model are used.

The Weber and Morris intraparticle diffusion model [73] is commonly expressed as:

(15) $$q_{t}=k_{\mathrm{intp}}\cdot t^{1/2}+C_{i}$$

where k_intp_ (mg/g·h^1/2^) is the intraparticle diffusion rate constant and Ci represents the effect of the extraparticle diffusion. These parameters can be obtained from the slope and the intercept of the line obtained by representing q_t_ against t^1/2^. For pure intraparticle diffusion to take place, that representation must be linear and pass through the origin. The presence of multilinearity in this representation means that the adsorption process is controlled by a combination of both extraparticle and intraparticle diffusion.

If this last case occurs, in order to distinguish the mainly controlling step of the adsorption process, the Boyd’s kinetic model [74] is usually employed. This model is described by the equation:

(16) $$B_{t}=-0.4977-\ln\left(1-\frac{q_{t}}{q_{e}}\right)$$

If the plot of B_t_ versus time is a straight line and passes through the origin, the adsorption process is mainly controlled by intraparticle diffusion; otherwise, it is mainly controlled by extraparticle diffusion.

**Figure 8:**
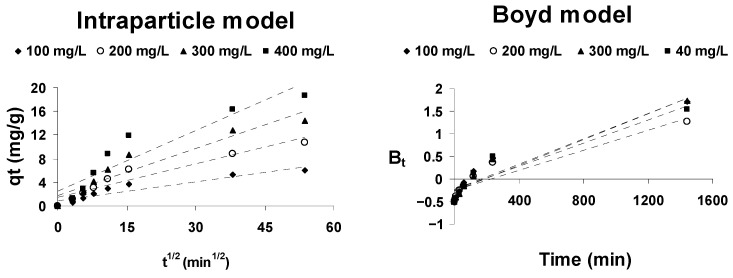
Adsorption mechanism plots for the adsorption of CPC onto AOS.

The mechanism of cephalosporin C adsorption onto activated olive stones has been analyzed by these two models. As can be seen from Figure 8, intraparticle model representations are not linear over the entire time range (multilinearity can be observed), which means that intraparticle diffusion is not the only adsorption rate-controlling step, but rather that there is more than one involved. That is, the dual nature of the intraparticle model graphs confirms that both extraparticle and intraparticle diffusion control CPC adsorption onto AOS.

The Boyd model representations are not linear and they do not pass through the origin, suggesting that the extraparticle (boundary layer) diffusion process is the step that mainly controls the rate of the adsorption process.

### 3.5. Thermodynamic Studies

#### 3.5.1. Effect of Temperature on Adsorption

It is well known that temperature greatly influences any adsorption process. Table 5 shows the values of the adsorption capacity of cephalosporin C onto the activated olive stones at different temperatures and different initial concentrations of cephalosporin C (the values of the pseudo-second-order rate constant at different temperatures are also shown).

It can be seen that an increase in temperature from 10 to 40 °C (283 K to 313 K) leads to an increase in adsorbent adsorption capacity for all initial CPC concentrations. This must be due both the decrease in the viscosity of the solution, which favors the mobility of the adsorbate to reach the surface and the interior of the pores of the adsorbent, and the increase in the interactions between the functional groups of CPC and AOS [75]. These results also indicate the endothermic character of the adsorption process of cephalosporin C onto activated olive stones.

#### 3.5.2. Thermodynamic Parameters

The thermodynamic parameters allow for evaluating the orientation, feasibility and possibilities of the application of an adsorption process. Therefore, the thermodynamic parameters standard free energy (ΔG^0^), standard enthalpy (ΔH^0^), standard entropy (ΔS^0^) and adsorption activation energy (E^a^) were determined from the following equations:

(17) $${\Delta G}^{0}=-R\cdot T\cdot {\mathrm{lnK}}_{e}$$

(18) $${\mathrm{lnK}}_{e}=-\frac{{\Delta G}^{0}}{R\cdot T}=-\frac{{\Delta H}^{0}}{R\cdot T}+\frac{{\Delta S}^{0}}{R}$$

(19) $$lnk_{2}=lnA-\frac{E^{a}}{R\cdot T}$$

where R is the universal gas constant, K_e_ is the equilibrium constant, k_2_ is the pseudo-second-order model rate constant, A is the Arrhenius factor and T is the absolute temperature (K). Values of K_e_ were calculated from the relation ln(q_e_/C_e_) versus q_e_ at different temperatures and extrapolating to zero [76].

**Figure 9:**
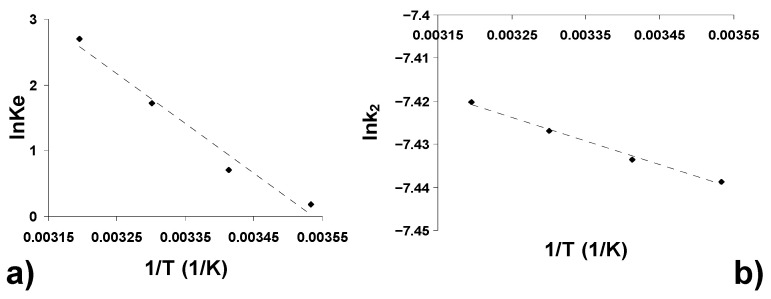
Thermodynamic of the adsorption of CPC onto AOE: (**a**) estimation of thermodynamic parameters; (**b**) estimation of the activation energy.

Plots of lnK_e_ and lnk_2_ versus 1/T (Figure 9a,b) should give straight lines with a slope of -∆H^0^/R and -E^a^/R, respectively, and an intercept of -∆S^0^/R and lnA, respectively.

The values of ΔG at different temperatures, ΔH^0^ and ΔS^0^ and E^a^, calculated from Equations (17)–(19), are shown in Table 6.

The negative values of ΔG for all the temperatures studied indicate that the adsorption process is spontaneous and thermodynamically favorable. The magnitude of these values (−20 kJ/mol < ΔG < 0 kJ/mol) shows the nature of the physiadsorption for the CPC-AOS system [77]. Increasing the negative value of ΔG with increasing temperature indicates the increase in adsorption with increasing temperature. The positive value of ΔH^0^ confirms the endothermic nature of the adsorption process. The positive value of ΔS^0^ theoretically confirms the affinity of AOS for CFC adsorption [78] and suggests the increase in the randomness of the solid/solution interface during the adsorption of CPC molecules on the adsorbent surface, probably due to structural changes in both the adsorbate and the adsorbent [79]. The low positive value of the activation energy confirms both the physical and endothermic nature of CPC adsorption on AOS [80].

### 3.6. Comparison with Other Adsorbents

In order to assess the possibilities of industrial competitive use of the activated olive stones in the removal of cephalosporin C from aqueous solutions, their efficiency, in terms of adsorption capacity (mol/kg), has been compared with that of active carbon and of other adsorbents described in the literature [81,82]. The results, shown in Table 7, indicate that the efficiency of AOS is 20% of that of activated carbon (the adsorbent which leads to the best results), 60% of that of the SP207 and SP850 adsorbents and it is about 65% higher than that of the XAD-2 adsorbent. These results allow us to affirm that the use activated olive stones as adsorbent for Cepalosporin C removal from aqueous solutions constitutes an interesting possibility from both economic and environmental points of view.

### 3.7. Practical Implications of This Study

The results obtained in this study allow us to affirm that the use of activated olive stones as an adsorbent for the elimination of antibiotics can be an interesting alternative to traditional adsorbents. Although its effectiveness, as indicated, is much lower than that of activated carbon (reference adsorbent), it is closer to and even exceeds that of other adsorbents. All this, together with its low price (if it has one), good chemical and mechanical resistance and eco-compatibility, makes olive stones a promising agro-industrial byproduct, from both economic and environmental points of view, to be used as an adsorbent for the elimination of emerging pollutants, although new research is necessary in order to further improve its adsorption capacity, looking for activation treatments that increase its surface area and improve its effectiveness against specific environmental problems.

## 4. Conclusions

The removal of cephalosprin C from aqueous solutions by adsorption on activated olive stones in a stirred tank was studied in this paper. A quantum study (molecular size, adsorbate–adsorbent molecular interactions, dipole moment and chemical reactivity predicted by the molecular orbitals) of the different existing species of cephalosporin C in aqueous solutions, depending on the pH, suggests a favorable mass transfer process during adsorption. SEM and EDX characterization of the adsorbent confirms that cephalosporin C is retained by activated olive stones. Both electrostatic and hydrogen bond interactions can be established between the adsorbate and the adsorbent at the pH of the adsorption experiments (HCl 10^−3^ M) as deduced from adsorbent pH_pzc_ value and the IR study. The adsorption process is best described by the Freundlich isotherm model and the pseudo-second-order kinetic model. Although more than one step is involved, the adsorption mechanism is mainly controlled by film diffusion. The adsorption process is of a physical nature, endothermic and spontaneous, under the conditions studied. Comparison of the results obtained in this paper with those obtained in other investigations shows that the efficiency of AOS is 20% of that of activated carbon, 60% of that of the SP207 and SP850 adsorbents, but 65% higher than that of the XAD-2 adsorbent. As the olive stone constitutes a highlyabundant and easily accessible agro-industrial by-product, with a very low price (if it has any), good chemical/mechanical resistance and eco-compatibility, its use as an adsorbent for the elimination of pollutants of emerging concern represents an interesting reality from both the economic and the environmental points of view.

## Figures and Tables

**Table 1:** Diedral angles of different ionizated forms of cephalosporin C.

Dihedrals Angles	CPC (Theor. Molec.)	CPCH_4_^2+^	CPCH_3_^+^	CPCH_2_	CPCH^−1^	CPC^−2^	
D(11,14,13,2)	46	52	55	54	55	49	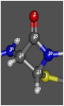
D(11,14,13,10)	−130	−127	−123	−123	−122	−128	
D(11,14,15,2)	169	172	174	174	174	171	
A(11,14,15)	117	114	113	113	113	117	
A(11,14,13)	121	119	118	119	118	121	

**Table 2:** Mulliken charge, molecular dipole, molar volume and total non-electrostatic energy of different forms of CPC.

Mulliken Charge	CPC (Theor. Molec.)	CPCH_4_^2+^	CPCH_3_^+^	CPCH_2_	CPCH^−1^	CPC^−2^
O (ring)	−0.043	−0.036	−0.042	−0.043	−0.572	−0.572
O (alkyl chain)	−0.070	−0.020	−0.024	−0.556	−0.557	−0.619
N (amide)	−0.136	0.309	−0.133	−0.135	−0.135	−0.295
N (amine)	−0.064	0.663	0.657	0.539	0.538	−0.139
Dipole (Debye)	2.443	40.618	3.504	15.924	30.138	30.671
HOMO Energy(eV)	−6.555	−6.914	−6.578	−6.316	−5.723	−5.430
LUMO Energy (ev)	−1.577	−2.039	−1.604	1.586	−0.673	−0.651
HOMO-LUMO Energy (eV)	−4.978	−4.875	−4.974	−4.730	−5.057	−4.779
Molar volumen (cm^3^/mol)	254.809	255.831	273.847	262.635	319.743	262.432
Total non-electrostatic (eV)	−0.82	−0.80	−0.81	−0.82	−0.86	−0.86

**Table 3:** Parameter value of the different studied isotherm models obtained in the adsorption of CPC onto AOS.

	**Langmuir Isotherm**				**Freundlich Isotherm**			**Elovich Isotherm**		
**Temperature (K)**	**q_m_ (mg/g)**	**K_L_ (L/mg)**	**R_L_**	**R^2^**	**N**	**K_F_ (mg/g)** **(L/mg)^1/n^**	**R^2^**	**q_mE_ (mg/g)**	**K_E_ (L/g)**	**R^2^**
283	31.25	0.00140	0.871	0.992	1.310	0.119	0.9992	21.65	0.0022	0.9927
293	55.56	0.00142	0.774	0.9576	1.261	0.180	0.9987	43.10	0.0019	0.9499
303	129.87	0.00168	0.665	0.8492	1.270	0.496	0.9940	102.04	0.0022	0.8014
313	227.27	0.00255	0.494	0.8745	1.257	1.153	0.9971	178.57	0.0033	0.8435
	**Temkin Isotherm**			**Javanovic Isotherm**			**Dubinin–Radushkevich Isotherm**			
**Temperature (K)**	**A (L/mg)**	**B (mg/g)**	**R^2^**	**q_m_ (mg/g)**	**K_J_ (L/mg)**	**R^2^**	**q_m_ (mg/g)**	**K (mol^2^/L^2^)**	**E (Jmol)**	**R^2^**
283	0.0213	5.082	0.9843	2.9755	0.0370	0.9506	10.07	1654.9	0.0174	0.9166
293	0.0224	8.649	0.9716	4.6464	0.0042	0.9557	16.68	1265.9	0.0199	0.9071
303	0.0271	19.892	0.9596	10.5804	0.0052	0.9729	37.81	790.39	0.0252	0.8688
313	0.0437	33.558	0.9502	17.2412	0.0083	0.9657	56.06	232.88	0.0463	0.9479

**Table 4:** Kinetics constants for the adsorption of CPC onto AOS at 20 °C.

		Pseudo-First Order			Pseudo-Second Order			Elovich			Avrami		
C_o_ (mg/L)	q_e,exp_	k_1_ (1/min)	q_e,cal_ (mg/g)	R^2^	k_2_ [g/(mg·min)]	q_e,cal_ (mg/g)	R^2^	A (mg/g·min)	B (g/mg)	R^2^	K_AV_ (1/min)	n_AV_	R^2^
100	5.9873	0.0014	4.5435	0.9164	0.00120099	6.1690	0.9981	0.1482	1.0097	0.9950	0.0294	0.6183	0.9811
200	10.6883	0.0011	8.2672	0.8923	0.00057981	10.9409	0.9934	0.2383	0.5828	0.9875	0.0352	0.5580	0.9877
300	14.3992	0.0014	11.4707	0.9317	0.00040596	15.0150	0.9985	0.2804	0.4017	0.9869	0.0200	0.6711	0.9860
400	18.7234	0.0013	39.2009	0.8810	0.00017744	20.3252	0.9958	0.3845	0.3115	0.9855	0.0196	0.6768	0.9643

**Table 5:** Temperature effect on the amount of CPC loaded onto the AOS (q_e_) at different CPC initial concentrations and on the rate constant of the pseudo-second-order adsorption kinetic model.

	Initial Cephalosporin C Concentration (mg/L)				
	100	200	300	400	Kinetic Constant k_2_ (g/mg·min)
Temparature (K)	q_e_ (mg/g)				
283	3.86	6.56	8.90	10.87	0.000588
293	5.99	10.69	14.41	18.72	0.000591
303	14.31	23.12	34.67	42.04	0.000595
313	21.82	40.71	57.58	76.42	0.000599

**Table 6:** Thermodynamic parameters of the adsorption of cephalosporin C onto AOS.

ΔH^0^ (kJ/mol)	ΔS^0^ (kJ/mol K)	ΔG (kJ/mol)				E^a^ (kJ/mol)
		283 K	293 K	303 K	313 K	
62.7618	0.2219	−0.0467	−2.2661	−4.4855	−6.7049	0.4569

**Table 7:** Cephalosporin C adsorption capacity onto different adsorbents.

Adsorbent [Ref.]	Temperature	pH	[CPC]0 mol/m^3^	q_e_ (mol/kg)
SP207 [81]	25 °C	5.3	5	0.061
			10	0.095
			15	0.123
			20	0.137
SP850 [82]	25 °C	7.5	5	0.032
			10	0.073
			15	0.119
			20	0.134
XAD-2 [81]	25 °C	5.3	5	0.018
			10	0.033
			15	0.045
			20	0.061
AC [this paper]	30 °C	HCl 10^−3^ M	0.209	0.161
			0.418	0.254
			0.627	0.358
			0.836	0.431
AOS [this paper]	30 °C	HCl 10^−3^ M	0.209	0.030
			0.418	0.048
			0.627	0.073
			0.836	0.088

## Data Availability

Results of this study are supported by the data included in the paper.

## References

[1] Russell V.S. (1974). Pollution: Concept and definition. Biol. Conserv..

[2] Norman Network Glossary of Terms: Emerging Pollutants. http://www.norman-network.net/?q=node/9#pos_E.

[3] Miller T.H., Bury N.R., Owen S.F., MacRae J.I., Barron L.P. (2018). A review of the pharmaceutical exposome in aquatic fauna. Environ. Pollut..

[4] Yu X., Sui Q., Lyu S., Zhao W., Liu J., Cai Z., Yu G., Barcelo D. (2020). Municipal solid waste landfills: An underestimated source of pharmaceutical and personal care products in the water environment. Environ. Sci. Technol..

[5] Yi X.Z., Lin C.H., Ong E.J.L., Wang M., Zhou Z. (2019). Occurrence and distribution of trace levels of antibiotics in surface waters and soils driven by non-point source pollution and anthropogenic pressure. Chemosphere.

[6] Bilal M., Mehmood S., Rasheed T., Iqbal H.M. (2020). Antibiotics traces in the aquatic environment: Persistence and adverse environmental impact. Curr. Opin. Environ. Sci. Health.

[7] Osińska A., Korzeniewska E., Harnisz M., Felis E., Bajkacz S., Jachimowicz P., Niestępski S., Konopka I. (2020). Small-scale wastewater treatment plants as a source of the dissemination of antibiotic resistance genes in the aquatic environment. J. Hazard. Mater..

[8] Bilal M., Ashraf S.S., Barceló D., Iqbal H.M. (2019). Biocatalytic degradation/redefining “removal” fate of pharmaceutically active compounds and antibiotics in the aquatic environment. Sci. Total Environ..

[9] Magdaleno A., Saenz M.E., Juarez A.B., Moretton J. (2015). Effects of six antibiotics and their binary mixtures on growth of *Pseudokirchneriella subcapitata*. Ecotoxicol. Environ. Saf..

[10] Pandei S., Sumant O. Cephalosporin Market by Generation (First-Generation, Second-Generation, Third-Generation, Fourth-Generation, and Fifth-Generation), Type (Branded and Generic), Route of Drug Administration (Intravenous and Oral), and Application (Respiratory Tract Infection, Skin Infection, Ear Infection, Urinary Tract Infection, and Sexually Transmitted Infection): Global Opportunity Analysis and Industry Forecast, 2019–2027. https://www.alliedmarketresearch.com/cephalosporin-market.

[11] Yu X., Tang X., Zuo J., Zhang M., Chen L., Li Z. (2016). Distribution and persistence of cephalosporins in cephalosporin producing wastewater using SPE and UPLC–MS/MS method. Sci. Total Environ..

[12] Ribeiro A.R., Bernd Sures B., Schmidt T.C. (2018). Cephalosporin antibiotics in the aquatic environment: A critical review of occurrence, fate, ecotoxicity and removal technologies. Environ. Pollut..

[13] El-Shaboury S.R., Saleh G.A., Mohamed F.A., Rageh A.H. (2007). Analysis of cephalosporin antibiotics. Rev. J. Pharm. Biomed. Anal..

[14] Sader H.S., Jones R.N. (1992). Historical overview of the cephalosporin spectrum: Four generations of structural evolution. Antimicrob. Newsl..

[15] Dąbrowska M., Muszyńska B., Starek M., Żmudzki P., Opoka W. (2018). Degradation pathway of cephalosporin antibiotics by in vitro cultures of Lentinula edodes and Imleria badia. Int. Biodeterior. Biodegrad..

[16] Guo R., Chen J. (2015). Application of alga-activated sludge combined system (AASCS) as a novel treatment to remove cephalosporins. Chem. Eng. J..

[17] Elbalkiny H.T., Yehia A., Riad S.M., Elsaharty T.S. (2019). Removal and tracing of cephalosporins in industrial wastewater by SPE-HPLC: Optimization of adsorption kinetics on mesoporous silica nanoparticles. J. Anal. Sci. Technol..

[18] Fakhri A., Adami S. (2014). Adsorption and thermodynamic study of Cephalosporins antibiotics from aqueous solution onto MgO nanoparticles. J. Taiwan Inst. Chem. Eng..

[19] Yang B., Zuo J., Li P., Wang K., Yu X., Zhang M. (2016). Effective ultrasound electrochemical degradation of biological toxicity and refractory cephalosporin pharmaceutical wastewater. Chem. Eng. J..

[20] Qian Y., Liu X., Li K., Gaoa P., Chen J., Liu Z., Zhou X., Zhang Y., Chen H., Li X. (2020). Enhanced degradation of cephalosporin antibiotics by matrix components during thermally activated persulfate oxidation process. Chem. Eng. J..

[21] Watkinson A.J., Murby E.J., Costanzo S.D. (2007). Removal of antibiotics in conventional and advanced wastewater treatment: Implications for environmental discharge and wastewater recycling. Water Res..

[22] Crini G., Lichtfouse E. (2019). Advantages and disadvantages of techniques used for wastewater treatment. Environ. Chem. Lett..

[23] Onaga-Medina F.M., Aguiar M.B., Parolo M.E., Avena M.J. (2021). Insights of competitive adsorption on activated carbon of binary caffeine and diclofenac solutions. J. Environ. Manag..

[24] Bedia J., Peñas-Garzón M., Gómez-Avilés A., Rodriguez J.J., Belver C. (2018). A Review on the Synthesis and Characterization of Biomass-Derived Carbons for Adsorption of Emerging Contaminants from Water. J. Carbon Res..

[25] Chen Y., Shi J., Du Q., Zhang H., Cui Y. (2019). Antibiotic removal by agricultural waste biochars with different forms of iron oxide. RSC Adv..

[26] Silva B., Martins M., Rosca M., Rocha V., Lago A., Neves I.C., Tavares T. (2020). Waste-based biosorbents as cost-effective alternatives to commercial adsorbents for the retention of fluoxetine from water. Sep. Purif. Technol..

[27] Lee C., Yang W., Parr R. (1988). Development of the Colle-Salvetti correlation-energy formula into a functional of the electron density. Phys. Rev. B.

[28] Becke A.D. (1993). Density-functional thermochemistry. III. The role of exact exchange. J. Chem. Phys..

[29] Frisch M.J., Trucks G.W., Schlegel H.B., Scuseria G.E., Robb M.A., Cheeseman J.R., Montgomery J.A., Vreven T., Kudin K.N., Burant J.C. (2004). Gaussian 03, Revision D.02.

[30] Tomasi J., Mennucci B., Cammi R. (2005). Quantum mechanical continuum solvation models. Chem. Rev..

[31] Mermer A., Bayrak H., Alyar S., Alagumuthu M. (2020). Synthesis, DFT calculations, biological investigation, molecular docking studies of β-lactam derivatives. J. Mol. Struct..

[32] Chern J.M., Wu C.Y. (2001). Desorption of dye from activated carbon beds: Effects of temperature, pH, and alcohol. Water Res..

[33] Calero de Hoces M., Blázquez-García G., Ronda-Gálvez A., Martín-Lara M.A. (2010). Effect of the Acid Treatment of Olive Stone on the Biosorption of Lead in a Packed-Bed Column. Ind. Eng. Chem. Res..

[34] Ahmad F., Daud W.M.A.W., Ahmad M.A., Radzi R. (2013). The effects of acid leaching on porosity and surface functional groups of cocoa (*Theobroma cacao*)-shell based activated carbon. Chem. Eng. Res. Des..

[35] León G., Hidalgo A.M., Miguel B., Guzmán M.A. (2020). Pertraction of Co(II) through Novel Ultrasound Prepared Supported Liquid Membranes Containing D2EHPA. Optimization and Transport Parameters. Membranes.

[36] Mahmood T., Saddique M.T., Naeem A., Westerhoff P., Mustafa S., Alum A. (2011). Comparison of Different Methods for the Point of Zero Charge Determination of NiO. Ind. Eng. Chem. Res..

[37] Blázquez G., Martín-Lara M.A., Dionisio-Ruiz E., Tenorio G., Calero M. (2011). Evaluation and comparison of the biosorption process of copper ions onto olive stone and pine bark. J. Ind. Eng. Chem..

[38] Tumampos S.B., Ensano B.M.B., Pingul-Ong S.M.B., Ong D.C., Kan C.C., Yee J.J., de Luna M.D.G. (2021). Isotherm, Kinetics and Thermodynamics of Cu(II) and Pb(II) Adsorption on Groundwater Treatment Sludge-Derived Manganese Dioxide for Wastewater Treatment Applications. Int. J. Environ. Res. Public Health.

[39] Molday R.S., Kallen R.G. (1972). Substituent effects on amide hydrogen exchange rates in aqueous solution. J. Am. Chem. Soc..

[40] Eriksson M.A.L., Hard T., Nilsson L. (1995). On the pH Dependence of Amide Proton Exchange Rates in Proteins. Biophys. J..

[41] Alekseev V.G. (2010). Acid–base properties of penicillins and cephalosporins (a review). Pharm. Chem. J..

[42] Ribeiro A.R., Schmidt T.C. (2017). Determination of acid dissociation constants (pKa) of cephalosporin antibiotics: Computational and experimental approaches. Chemosphere.

[43] Zhang K., Cassady C.J., Chung-Phillips A. (1994). Ab Initio Studies of Neutral and Protonated Triglycines: Comparison of Calculated and Experimental Gas-Phase Basicity. J. Am. Chem. Soc..

[44] Naja G., Mustin C., Volesky B., Berthelin J. (2005). A high resolution titrator: A new approach to studying binding sites of microbial biosorbents. Water Res..

[45] Deng S., Ting Y.P. (2005). Characterization of PEI-modified biomass and biosorption of Cu(II), Pb(II) and Ni(II). Water Res..

[46] Martínez M., Miralles N., Hidalgo S., Fiol N., Villaescusa I., Poch J. (2006). Removal of lead(II) and cadmium(II) from aqueous solutions using grape stalk waste. J. Hazard. Mater..

[47] Bohli T., Ouederni A., Fiol N., Villaescusa I. (2015). Evaluation of an activated carbon from olive stones used as an adsorbent for heavy metal removal from aqueous phases. Comptes Rendus Chim..

[48] Benzekri M.B., Benderdouche N., Bestani B., Douara N., Duclaux L. (2018). Valorization of olive stones into a granular activated carbon for the removal of methylene blue in batch and fixed bed modes. J. Mater. Environ. Sci..

[49] Fiol N., Villaescusa I. (2009). Determination of sorbent point zero charge: Usefulness in sorption studies. Environ. Chem. Lett..

[50] Langmuir I. (1918). The adsorption of gases on plane surfaces of glass, Mica and platinum. J. Am. Chem. Soc..

[51] Wang J., Guo X. (2020). Adsorption isotherm models: Classification, physical meaning, application and solving method. Chemosphere.

[52] Zaheer Z., Aisha A.A., Aazam E.S. (2019). Adsorption of methyl red on biogenic Ag@Fe nanocomposite adsorbent: Isotherms, kinetics and mechanisms. J. Mol. Liq..

[53] Weber T., Chakravorti R. (1974). Pore and solid diffusion models for fixed-bed adsorbers. AIChE J..

[54] Freundlich H. (1906). Over the adsorption in solution. J. Phys. Chem..

[55] Foo K.Y., Hameed B.H. (2010). Insights into the modeling of adsorption isotherm systems. Chem. Eng. J..

[56] Al-Ghouti M.A., Da’ana D.A. (2020). Guidelines for the use and interpretation of adsorption isotherm models: A review. J. Hazard. Mater..

[57] León G., García F., Miguel B., Bayo J. (2016). Equilibrium, kinetics and thermodynamic studies of methyl orange removal by adsorption onto granular activated carbon. Desalin. Water Treat..

[58] Temkin M., Pyzhev V. (1940). Kinetics of Ammonia synthesis on promoted Iron catalysts. Acta Physicochim. URSS.

[59] Rajahmundry G.K., Garlapati C., Kumar P.S., Alwi R.S., Vo D.V.N. (2021). Statistical analysis of adsorption isotherm models and its appropriate selection. Chemosphere.

[60] Jovanovic D.S. (1969). Physical adsorption of gases. I: Isotherms for monolayer and multilayer adsorption. Kolloid Z. Z. Polym..

[61] Saadi R., Saadi Z., Fazaeli R., Fard N. (2015). Monolayer and multilayer adsorption isotherm models for sorption from aqueous media. Korean J. Chem. Eng..

[62] Dubinin M.M., Radushkevich L.V. (1947). The equation of the characteristic curve of the activated charcoal. Proc. Acad. Sci. Ussr Phys. Chem. Sect..

[63] Dubinin M. (1960). The potential theory of adsorption of gases and vapors for adsorbents with energetically non-uniform surfaces. Chem. Rev..

[64] Lagergren S. (1898). About the theory of so-called adsorption of soluble substances. K. Sven. Vetensk. Handl..

[65] Tseng R.L., Wu F.C., Juang R.S. (2010). Characteristics and applications of the Lagergren’s first-order equation for adsorption kinetics. J. Taiwan Inst. Chem. Eng..

[66] Ho Y.S. (2006). Review of second-order model for adsorption systems. J. Hazard. Mater..

[67] Hubbe M.A., Azizian S., Douven S. (2019). Implications of apparent pseudo-second-order adsorption kinetics onto cellulosic materials: A review. BioResources.

[68] Elovich S.Y., Larinov O.G. (1962). Theory of adsorption from solutions of non-electrolytes on solid (I) equation adsorption from solutions and the analysis of its simplest form, (II) verification of the equation of adsorption isotherm from solutions. Izvestyja Akad. Nauk SSSR Otd. Khimicheskikh Nauk.

[69] Chien S.H., Clayton W.R. (1980). Application of Elovich equation to the kinetics of phosphate release and sorption in soils. Soil Sci. Soc. Am. J..

[70] Avrami M. (1940). Kinetics of phase change. I General theory. J. Chem. Phys..

[71] Samiey B., Abdollahi-Jonaghani S. (2015). A New Approach for Analysis of Adsorption from Liquid Phase: A Critical Review. J. Pollut. Eff. Control.

[72] Acharya J., Sahu J.N., Sahoo B.K., Mohanty C.R., Meikap B.C. (2009). Removal of chromium(VI) from wastewater by activated carbon developed from Tamarind wood activated with zinc chloride. Chem. Eng. J..

[73] Weber W.J., Morris J.C. (1963). Kinetics of adsorption on carbon from solution. J. Sanit. Eng. Div..

[74] Boyd G.E., Adamson A.W., Myers L.S. (1947). The exchange adsorption of ions from aqueous solutions by organic zeolites. II. Kinetics. J. Am. Chem. Soc..

[75] Tabak A., Baltas N., Afsin B., Emirik M., Caglar B., Eren E. (2010). Adsorption of Reactive Red 120 from aqueous solutions by cetylpyridinium-bentonite. J. Chem. Technol. Biotechnol..

[76] Amin N.K. (2009). Removal of direct blue-106 dye from aqueous solution using new activated carbons developed from pomegranate peel: Adsorption equilibrium and kinetics. J. Hazard. Mater..

[77] Weng C.H., Lin Y.T., Tzeng T.W. (2009). Removal of methylene blue from aqueous solution by adsorption onto pineapple leaf powder. J. Hazard. Mater..

[78] Zhang J., Cai D., Zhang G., Cai C., Zhang C., Qiu G., Zheng K., Wu Z. (2013). Adsorption of methylene blue from aqueous solution onto multiporous palygorskite modified by ion beam bombardment: Effect of contact time, temperature, pH and ionic strength. Appl. Clay Sci..

[79] Agarwal S., Tyagi I., Gupta V.K., Ghasemi N., Shahivand M., Ghasemi M. (2016). Kinetics, equilibrium studies and thermodynamics of methylene blue adsorption on Ephedra strobilacea saw dust and modified using phosphoric acid and zinc chloride. J. Mol. Liq..

[80] Vasiliu S., Bunia I., Racovita S., Neagu V. (2011). Adsorption of cefotaxime sodium salt on polymer coated ion exchange resin microparticles: Kinetics, equiklibrium and thermodynamic studies. Carbohydr. Polym..

[81] Lee J.W., Park H.C., Moon H. (1997). Adsorption and desorption of cephalosporin C on non-ionic polymeric sorbents. Sep. Purif. Technol..

[82] Lee J.W., Moon H. (1999). Effect of pH on Adsorption of Cephalosporin C by A Nonionic Polymeric Sorbent. Adsorption.

